# Trends in self-reported current diabetes care use, socioeconomic inequalities, and associated factors in Japan: evidence from the Comprehensive Survey of Living Conditions (1986–2022)

**DOI:** 10.1136/bmjdrc-2026-006042

**Published:** 2026-07-15

**Authors:** Tshewang Gyeltshen, Hirokazu Tanaka, Kota Katanoda

**Affiliations:** 1 Division of Population Data Science, National Cancer Center Institute for Cancer Control, Tokyo, Japan; 2 Department of Global Health Policy, School of International Health, The University of Tokyo, Tokyo, Japan

**Keywords:** Epidemiology, Public Health, Diabetes Mellitus, Type 2, Diabetes Mellitus, Type 1

## Abstract

**Introduction:**

Long-term trends in diabetes care use and socioeconomic inequalities are not well described in Japan.

**Research design and methods:**

We analyzed nationally representative data from the Comprehensive Survey of Living Conditions (1986–2022). Diabetes care use was defined as self-reported current medical facility attendance for diabetes. Age-standardized prevalence was calculated as the proportion of adults currently receiving outpatient diabetes care; undiagnosed cases and diagnosed individuals not currently in treatment were therefore not captured. In the 2022 survey, a survey-weighted Poisson regression model was used to estimate prevalence ratios (PRs) of factors associated with current diabetes care use, including educational level and occupational class.

**Results:**

Age-standardized self-reported current diabetes care use increased from 1986 to 2022, rising from 2.2% to 8.3% in men and from 1.7% to 4.4% in women, with a significant widening of the sex inequalities over the study period. Educational differences were evident, particularly among working-age adults. In 2022, compared with the high-education group, PRs were 1.17 (95% CI 1.13 to 1.22) for the middle-education group and 1.22 (95% CI 1.13 to 1.32) for the low-education group for both sexes. Occupational differences were more modest. Compared with upper non-manual workers, lower non-manual workers had a lower PR=0.92 (95% CI 0.86 to 0.98), while non-employed individuals had a higher PR=1.10 (95% CI 1.01 to 1.21); manual workers, self-employed individuals, and farmers did not differ significantly from the reference group. Older age, men, hypertension, depression, and current smoking were also associated with higher current diabetes care use.

**Conclusions:**

Self-reported diabetes care use increased from 1986 to 2022 in Japan, with lower educational level associated with higher current diabetes care use, particularly among working-age adults. These findings help inform continued monitoring of socioeconomic inequalities in diabetes care.

**WHAT IS ALREADY KNOWN ON THIS TOPIC**

- The global rise in diabetes, including in Japan, poses a major public health challenge in high-income countries, with aging populations owing to severe complications.

**WHAT THIS STUDY ADDS**

- From 1986 to 2022, the prevalence of self-reported diabetes care use in Japan rose significantly. Analyses of the 2022 wave reveal clear educational inequalities concentrated among working-age adults, while the educational gradient was less consistent among older men. These patterns emphasize the growing burden of diabetes in an aging society and the need for long-term monitoring across generations.

**HOW THIS STUDY MIGHT AFFECT RESEARCH, PRACTICE OR POLICY**

- These findings highlight the importance of continued monitoring of socioeconomic differences in diabetes care in Japan and can help guide more targeted public health interventions. The Comprehensive Survey of Living Conditions offers valuable insights into long-term trends and should complement other surveys such as the National Health and Nutrition Survey in Japan.

## Introduction

 The rising global prevalence of diabetes is a major public health challenge, including in Japan, and the disease causes severe complications such as cardiovascular disease, kidney failure, neuropathy, and retinopathy.^1^,^4^ Lower socioeconomic status (SES) is a key determinant of diabetes risk through unfavorable lifestyle factors, poor healthcare access, and other adverse social conditions,^5^,^8^ disproportionately affecting vulnerable populations.

Three outcome concepts are often conflated: (1) biomarker-defined or ‘suspected’ diabetes (eg, HbA1c or fasting glucose); (2) diagnosed diabetes, regardless of treatment status; and (3) current diabetes care use, capturing only those actively engaged with the care system. These measures overlap but are not identical: biomarker surveys detect cases missed by self-report, and diagnosed-but-untreated individuals are absent from care use measures.

In Japan, about 30% of the population is aged 65 years or older, exacerbating diabetes morbidity.^9^ The 2024 National Health and Nutrition Survey (NHNS) reported that 12.9% of Japanese adults (20 years and older) (17.7% of men and 9.3% of women) have diabetes (‘strongly suspected’), corresponding to more than 11 million people. Of those previously diagnosed, 67.4% were currently receiving treatment (73.1% of men and 60.5% of women); the highest untreated proportions were among adults aged 30–49 years.^10^ Despite universal healthcare coverage since the 1960s, inequalities in non-communicable diseases such as diabetes persist across socioeconomic strata.^11^

Earlier studies on socioeconomic inequalities in diabetes have focused on selected subpopulations or specific sources such as hospital outpatient or insurance claims data.^11 12^ National annual surveys such as the NHNS report biomarker and treatment indicators but lack the long-term repeated cross-sectional data on current diabetes care use and its socioeconomic patterning, which are available in the Comprehensive Survey of Living Conditions (CSLC), making the CSLC a valuable complementary source for monitoring long-term trends in the general Japanese population.

Using the CSLC, this study aims to (1) examine secular trends in self-reported current diabetes care use in Japan from 1986 to 2022, (2) characterize socioeconomic inequalities by educational level (available from 2010 onward) and occupational class, and (3) assess associated factors.

## Methods

### Study design, settings, and data sources

We analyzed repeated cross-sectional data from the CSLC, a nationally representative population-based survey conducted every 3 years from 1986 to 2022 by Japan’s Ministry of Health, Labour and Welfare (MHLW). The CSLC samples approximately 500 000–800 000 people per survey wave across all prefectures and collects data on family composition, occupation, income, and self-rated health. Anonymized microdata were obtained from the MHLW under Article 33 of Japan’s Statistics Act.

## Study variables

### Definition of diabetes and related outcome

Self-reported diabetes was identified using Question 4 of the CSLC survey, which asked, “Are you currently visiting a hospital or clinic?” Respondents who answered ‘Yes’ and selected the code for ‘diabetes’ in the corresponding supplementary list of conditions in Question 4-1 were classified as currently receiving care for diagnosed diabetes. Accordingly, in this study, we refer to this outcome as ‘self-reported diabetes care use’. It is a lower bound on self-reported prevalence, excluding (1) diagnosed individuals not in treatment at the time of the survey and (2) undiagnosed cases. The CSLC does not distinguish type 1 from type 2 diabetes. In Japan, type 1 accounts for about 6% of adults with treated diabetes^13^ (incidence ~4.4 per 100 000 person-years^14^); the outcome therefore predominantly reflects treated type 2 diabetes.

### Socioeconomic indicators

SES was assessed using occupational class and educational level. Occupational class was categorized into five groups according to the modified Erikson-Goldthorpe-Portocarero (EGP) scheme: upper non-manual workers, lower non-manual workers, manual workers, farmers, and the self-employed, as in a previous study.^15^ Occupations not classified within the EGP scheme (eg, security workers and non-employees) were added in the regression analysis as separate categories so that none were excluded from the regression on the basis of occupation alone.^15^

Educational level (available from 2010) was grouped into three International Standard Classification of Education (ISCED) categories: ‘low’ (ISCED levels 1–2; elementary or junior high school), ‘middle’ (ISCED levels 3–4; high school or technical/professional school), and ‘high’ (ISCED levels 5–8; 2-year college, university, or graduate school).^15^

### Covariates

All covariates were self-reported in the 2022 CSLC health and household modules. In addition to sex and 5-year age categories, the following covariates were included: marital status (married (reference), single, widowed, divorced); household composition (living alone, more than one (reference)); health insurance (employees’ health insurance (reference), national health insurance, medical care system for the elderly aged 75 years and over, and others); hypertension and depression (defined in the same manner as the diabetes outcome); smoking (current, never/former (reference)); at-risk drinking as per Health Japan 21 (≥40 g/day ethanol for men, ≥20 g/day for women); and health screening participation in the prior year (yes vs no (reference)).^16^

### Statistical analysis

We calculated crude and age-standardized prevalence of self-reported diabetes care use among adults aged 20–94 years between 1986 and 2022, using direct standardization with the 2015 Japan Standard Population (5-year age categories).^17^ We examined self-reported diabetes care use by occupational class (25–64 years) and educational level (25–64 years and 65–94 years). All analyses were performed using weights derived from the MHLW weighting scores. Occupational class was restricted to ages 25–64 years because it primarily reflects current paid employment; most adults aged 65 years and older in Japan have exited the workforce. Educational level was presented separately for adults aged 25–64 years and 65–94 years because the educational distribution differs markedly between these birth cohorts, and because the absolute prevalence of diabetes care use differs substantially between working-age and older adults. To test whether the sex inequalities changed over time, we fitted a survey-weighted Poisson regression on pooled data from all 13 CSLC waves (1986–2022), adjusted for 5-year age categories, with centered survey year, sex, and a year×sex interaction, clustered at the prefecture level.

Associations between socioeconomic indicators and diabetes care use were examined with a survey-weighted Poisson model (log link, robust variance) on the 2022 wave. Educational level was unavailable before 2010, and several covariates were not comparable across earlier waves, so we did not pool waves for the multivariable analysis. The covariates were age (5-year categories), sex, marital status, household composition, health insurance, hypertension, depression, smoking, alcohol use, health screening, educational level, and occupational class. As a sensitivity analysis, we fitted a mixed-effects logistic regression with prefecture-level random intercepts to assess whether the direction and significance of associations were consistent under an alternative framework. Because ORs from a logistic model are not directly comparable to prevalence ratios (PR) for non-rare outcomes, this analysis was interpreted as a check on direction and approximate significance.

To assess whether the 2022 associations were wave specific, we refit the primary Poisson model in each wave with educational level (2010, 2013, 2016, 2019, and 2022), holding model specification, reference categories, link function, and prefecture-level clustering constant. A harmonized covariate set (age, sex, hypertension, depression, smoking, alcohol, health screening, educational level, and occupational class) was used for cross-wave comparability; marital status, household composition, and health insurance were omitted because they were not uniformly available for this analysis across waves, and alcohol was omitted for 2010 (not collected). Estimates are adjusted cross-sectional associations and not causal effects.^18^

### Handling of missing data

The primary multivariable analyses used complete-case analysis. Multiple imputation was not performed because missingness was concentrated in two structural variables (educational level and occupational class), where the missing-not-at-random assumption could not be ruled out. ‘Unknown’ educational level (retained in descriptive table 1 and online supplemental eTable 1) was treated as missing in regressions. For occupational class, a separate source data ‘occupation unknown’ group was retained as its own category and reported in table 2.

**Table 1:** Number of survey participants* by occupational class and educational levels in selected years, the Comprehensive Survey of Living Conditions, 1986–2022

	Survey year	1986 n (%)	2004 n (%)	2013 n (%)	2022 n (%)
**Men**					
All population (aged 25–64 years)		206 534	151 393	146 310	101 954
Occupational class (EGP scheme, aged 25–64 years)					
	Upper non-manual workers (I+II)	26 419 (12.8)	35 196 (23.2)	41 682 (28.5)	30 517 (29.9)
	Lower non-manual workers (III)	50 985 (24.7)	30 009 (19.8)	30 480 (20.8)	20 414 (20.0)
	Manual workers (V+VI+ VIIa)	59 409 (28.8)	32 630 (21.6)	28 760 (19.7)	22 635 (22.2)
	Farmers (IVc+VIIb)	19 387 (9.4)	4712 (3.1)	4363 (3.0)	2612 (2.6)
	Self-employed (IVa+b)	29 460 (14.3)	16 754 (11.1)	11 582 (7.9)	6471 (6.3)
	Other occupations not classified as EGP scheme (eg, security workers)	3296 (1.6)	3303 (2.2)	2652 (1.8)	1884 (1.8)
	Unknown	1491 (0.7)	2828 (1.9)	3765 (2.6)	3702 (3.6)
	Non-employee	16 087 (7.8)	25 961 (17.1)	23 026 (15.7)	13 719 (13.5)
Educational level (aged 25–64 years)					
	Low (ISCED: 1–2)	N/A	N/A	10 963 (7.5)	4538 (4.5)
	Middle (ISCED: 3–4)			72 759 (49.7)	46 930 (46.0)
	High (ISCED: 5–8)			47 785 (32.7)	37 086 (36.4)
	Unknown			14 803 (10.1)	13 400 (13.1)
Educational level (aged 65–94 years)					
	Low (ISCED: 1–2)	N/A	N/A	20 216 (31.1)	12 167 (18.0)
	Middle (ISCED: 3–4)			25 679 (39.4)	27 174 (40.1)
	High (ISCED: 5–8)			9756 (15.0)	14 541 (21.5)
	Unknown			9454 (14.5)	13 864 (20.5)
**Women**					
All population (aged 25–64 years)		218 975	158 703	153 604	105 474
Occupational class (EGP scheme, aged 25–64 years)					
	Upper non-manual workers (I+II)	12 227 (5.6)	14 613 (9.2)	24 544 (16.0)	21 348 (20.2)
	Lower non-manual workers (III)	48 132 (22.0)	33 008 (20.8)	53 818 (35.0)	40 227 (38.1)
	Manual workers (V+VI+ VIIa)	28 037 (12.8)	9207 (5.8)	10 513 (6.8)	8103 (7.7)
	Farmers (IVc+VIIb)	15 309 (7.0)	1983 (1.2)	2529 (1.6)	1434 (1.4)
	Self-employed (IVa+b)	7003 (3.2)	3569 (2.2)	3485 (2.3)	2385 (2.3)
	Other occupations not classified as EGP scheme (eg, security workers)	108 (0.1)	374 (0.2)	125 (0.1)	138 (0.1)
	Unknown	1309 (0.6)	2539 (1.6)	3978 (2.6)	3905 (3.7)
	Non-employee	106 850 (48.8)	93 410 (58.9)	54 612 (35.6)	27 934 (26.5)
Educational level (aged 25–64 years)					
	Low (ISCED: 1–2)	N/A	N/A	8517 (5.5)	2741 (2.6)
	Middle (ISCED: 3–4)			83 180 (54.2)	50 783 (48.1)
	High (ISCED: 5–8)			46 231 (30.1)	38 086 (36.1)
	Unknown			15 676 (10.2)	13 864 (13.1)
Educational level (aged 65–94 years)					
	Low (ISCED: 1–2)	N/A	N/A	30 884 (37.1)	17 487 (21.5)
	Middle (ISCED: 3–4)			35 363 (42.5)	37 671 (46.2)
	High (ISCED: 5–8)			4843 (5.8)	9453 (11.6)
	Unknown			12 102 (14.5)	16 844 (20.7)

* Number of survey participants across the selected years.

EGP scheme, Erikson-Goldthorpe-Portocarero scheme; ISCED, International Standard Classification of Education.

**Table 2:** Factors associated with self-reported diabetes care use in Japan, 2022

Characteristics		Survey-weighted Poisson model, model 1 (primary model)		
		PR	95% CI	P value
Age (years)				
	25–34	1.00	Reference	
	35–44	3.08	(2.35 to 4.05)	<0.001
	45–54	7.57	(5.67 to 10.11)	<0.001
	55–64	14.21	(11.00 to 18.33)	<0.001
	65–74	19.42	(15.27 to 24.70)	<0.001
	75–84	17.00	(12.43 to 23.21)	<0.001
	85–94	13.85	(9.63 to 19.93)	<0.001
Sex				
	Women	1.00	Reference	
	Men	1.93	(1.84 to 2.03)	<0.001
Marital status				
	Married	1.00	Reference	
	Single	1.14	(1.04 to 1.24)	0.006
	Widowed	1.08	(1.02 to 1.15)	0.017
	Divorced	1.02	(0.95 to 1.10)	0.589
Household				
	More than one	1.00	Reference	
	Living alone	0.96	(0.89 to 1.02)	0.067
Health insurance				
	Employees’ health insurance	1.00	Reference	
	National health insurance	1.05	(0.99 to 1.11)	0.077
	Medical care system for the elderly aged 75 years and over	1.23	(1.02 to 1.49)	0.034
	Others	1.49	(1.30 to 1.70)	<0.001
Hypertension				
	No	1.00	Reference	
	Yes	1.79	(1.74 to 1.85)	<0.001
Depression				
	No	1.00	Reference	
	Yes	1.40	(1.28 to 1.53)	<0.001
Smoking				
	No	1.00	Reference	
	Yes	1.07	(1.03 to 1.12)	<0.001
Alcohol				
	No	1.00	Reference	
	Yes	0.75	(0.71 to 0.79)	<0.001
Health screening				
	No	1.00	Reference	
	Yes	0.89	(0.85 to 0.92)	<0.001
Educational level				
	High (eg, university)	1.00	Reference	
	Middle (eg, high school)	1.17	(1.13 to 1.22)	<0.001
	Low (eg, junior high school)	1.22	(1.13 to 1.32)	<0.001
Occupational class				
	Upper non-manual workers (I+II)	1.00	Reference	
	Lower non-manual workers (III)	0.92	(0.86 to 0.98)	0.014
	Manual workers (V+VI+ VIIa)	0.89	(0.77 to 1.01)	0.070
	Self-employed (IVa+b)	1.00	(0.89 to 1.13)	0.973
	Farmers (IVc+VIIb)	0.88	(0.76 to 1.02)	0.080
	Other occupations not classified as EGP scheme	1.15	(0.94 to 1.42)	0.167
	Unknown	0.93	(0.81 to 1.07)	0.285
	Non-employee	1.10	(1.01 to 1.21)	0.038

EGP scheme, Erikson-Goldthorpe-Portocarero scheme; ISCED, International Standard Classification of Education; PR, prevalence ratio.

### Analytic samples and exclusions

**Figure 1:**
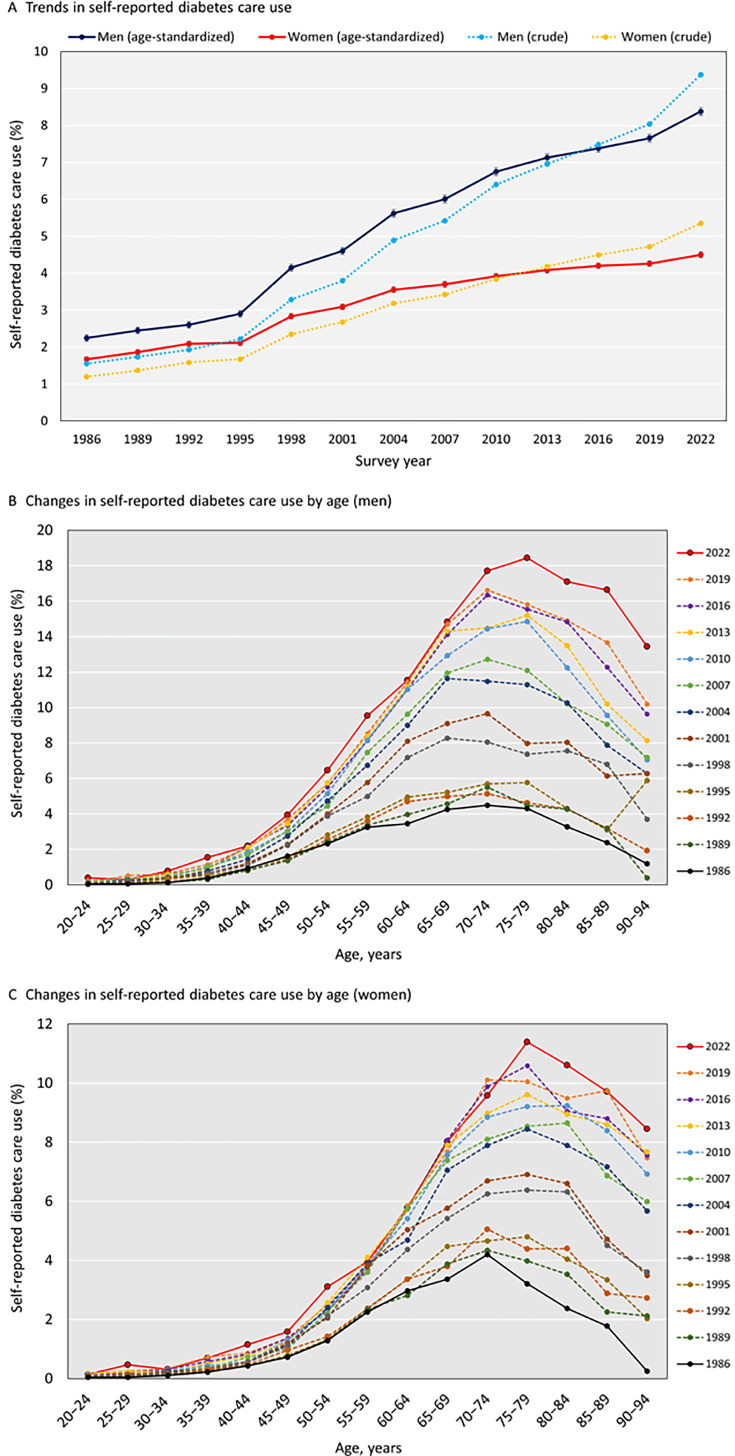
Trends in self-reported current diabetes care use among Japanese men and women aged 20–94 years, 1986–2022: (A) Trends in self-reported diabetes care use, (B) Changes in self-reported diabetes care use by age (men), (C) Changes in self-reported diabetes care use by age (women).

**Figure 2:**
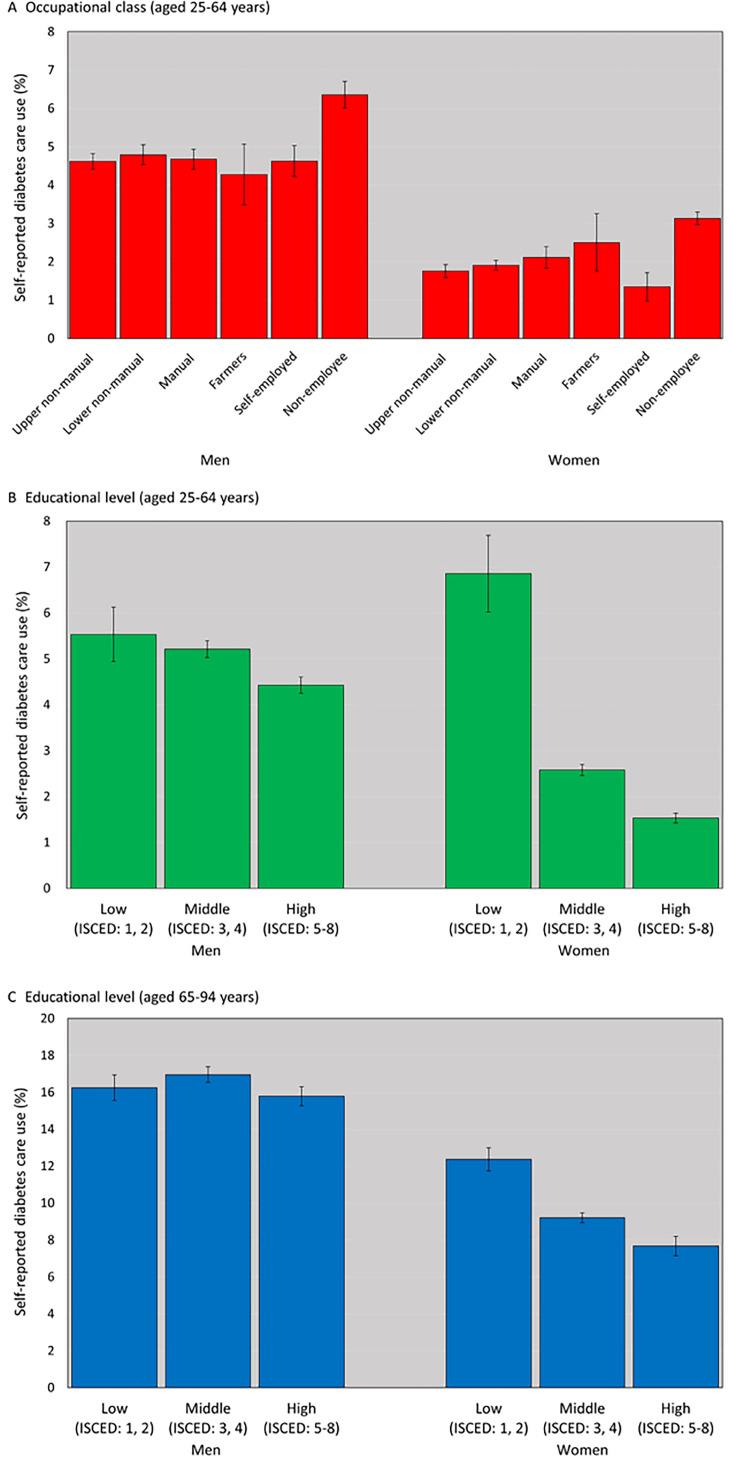
Self-reported current diabetes care use across occupational class and educational level by sex and age group in 2022: (A) Occupational class (aged 25–64 years), (B) Educational level (aged 25–64 years), (C) Educational level (aged 65–94 years). ISCED, International Standard Classification of Education.

Different components of the analysis used different samples. Trend analyses (figure 1) included all respondents aged 20–94 years in each wave from 1986 to 2022. Descriptive distributions by SES (table 1, online supplemental eTable 1, figure 2) presented age-stratified distributions of education (from 2010 to 2022) and occupational class for adults aged 25–64 years (occupation and younger education stratum) and 65–94 years (older education stratum), with missing or non-classifiable values retained as ‘Unknown’. The multivariable model (table 2) was restricted to 2022 respondents aged 25 years and older (n=376 573), with a total sample of 293 718 (78.0%) after complete-case deletion. The wave-by-wave sensitivity analysis (online supplemental eTable 2) used per-wave complete-case samples under a harmonized covariate set (2010, n=345 927; 2013, n=390 696; 2016, n=356 573; 2019, n=335 695; 2022, n=295 680), with the 2022 sample differing from the primary sample because marital status, household composition, and health insurance were omitted from the harmonized set (see online supplemental eTable 2 footnote).

All statistical analyses were conducted using Stata V.17/MP (StataCorp, College Station, Texas, USA) and R V.4.5.1 (R Foundation for Statistical Computing, Vienna, Austria) using the survey and lme4 packages. Statistical significance was defined as a two-sided p value <0.05.

## Results

### Characteristics of study participants

Table 1 presents distributions of survey participants by sex, occupational class, and educational level (full series 1986–2022 in (online supplemental eTable 1). SES shifted toward higher occupational class and educational levels across waves.

### Trends of self-reported diabetes care use (1986–2022)

Figure 1A shows long-term trends in self-reported current diabetes care use among men and women aged 20–94 years from 1986 to 2022. Both crude and age-standardized proportions rose in both sexes, with men consistently higher. Age-standardized prevalence increased from 2.2% to 8.3% in men and from 1.7% to 4.4% in women, widening the absolute sex gap from 0.5 to 3.9 percentage points. Figure 1B,C show sex and age-specific trends. Care use increased markedly with age in both sexes (especially 50 years and older), with men higher than women across nearly all age groups and waves (eg, in 2022, 17.7% of men vs 9.6% of women aged 70–74 years), although the relative increase was steeper for women in some older groups. Among adults under 40 years, prevalence remained below 1% throughout the period in both sexes. A pooled survey-weighted Poisson regression across all 13 waves (1986–2022), adjusted for 5-year age categories and clustered at the prefecture level, confirmed that this inequality widened significantly: the male to female PR of self-reported current diabetes care use increased by approximately 7.4% per decade (year×sex interaction: exp(β)=1.0072 per year, 95% CI 1.0062 to 1.0083, p<0.001; full model results in online supplemental eTable 3).

### Socioeconomic inequalities in 2022

Figure 2A shows self-reported diabetes care use by occupational class in 2022. Among working-age men (25–64 years), prevalence varied modestly across categories. Among working-age women, prevalence was lower overall (1.3–2.5%), highest among farmers (2.5%) and lowest among the self-employed (1.3%).

Figure 2B,C show diabetes care use by educational level. An inverse education gradient was evident in most subgroups but less consistent among older men. Among working-age men (25–64 years), diabetes care use fell from 5.5% in the low-education group to 4.4% in the high-education group; among older men (65–94 years), it was highest in the middle group (17.0%), followed by low (16.3%) and high (15.8%). Among women, the gradient was clearer: 6.9% (low) to 1.5% (high) for 25–64 years and 12.4% (low) to 7.7% (high) for 65–94 years.

### Factors associated with self-reported diabetes care use (the 2022 survey)

Table 2 summarizes factors associated with self-reported current diabetes care use in 2022. Diabetes care use increased progressively with age, peaking at 65–74 years (PR=19.4, 95% CI 15.3 to 24.7), compared with the 25–34 age group. Men were nearly twice as likely as women (PR=1.93, 95% CI 1.84 to 2.03).

Single (PR=1.14, 95% CI 1.04 to 1.24) and widowed individuals (PR=1.08, 95% CI 1.02 to 1.15) had slightly higher care use than married individuals; divorced status and living alone showed no significant difference (living alone; PR=0.96, 95% CI 0.89 to 1.02). Beneficiaries of the medical care system for the elderly aged 75 and over had higher care use than those with employees’ health insurance (PR=1.23, 95% CI 1.02 to 1.49); national health insurance showed no significant difference.

Hypertension was among the strongest correlates (PR=1.79, 95% CI 1.74 to 1.85), followed by depression (PR=1.40, 95% CI 1.28 to 1.53). At-risk drinking was associated with lower care use (PR=0.75, 95% CI 0.71 to 0.79) and current smoking with slightly higher use (PR=1.07, 95% CI 1.03 to 1.12). Recent health screening was associated with lower care use (PR=0.89, 95% CI 0.85 to 0.92).

Educational level remained independently associated with diabetes care use. In comparison to high education, middle (PR=1.17, 95% CI 1.13 to 1.22) and low (PR=1.22, 95% CI 1.13 to 1.32) educational levels had higher diabetes care use. Occupational differences were modest. Compared with upper non-manual workers, lower non-manual workers had lower diabetes care use (PR=0.92, 95% CI 0.86 to 0.98) and non-employed individuals slightly higher (PR=1.10, 95% CI 1.01 to 1.21); manual workers, farmers, and the self-employed showed no significant difference. The mixed-effects sensitivity analysis (online supplemental eTable 4) was directionally consistent, though some borderline associations reached significance under the alternative model. Retaining ‘Unknown’ as an explicit education category (online supplemental eTable 5) yielded similar PRs for middle (1.18, 95% CI 1.14 to 1.23) and low (1.26, 95% CI 1.16 to 1.36) versus high education, indicating the educational gradient was not materially affected by complete-case exclusion.

Wave-by-wave re-estimation across 2010–2022 (online supplemental eTable 2) was consistent with the 2022 results. The education gradient was stable: PRs ranged from 1.11 to 1.19 (middle vs high) and from 1.20 to 1.28 (low vs high) across all five waves. Men (from 1.87 to 1.93), hypertension (from 1.78 to 1.84), and non-employment (from 1.15 to 1.22) were similarly consistent, indicating that the 2022 patterns reflect a sustained rather than wave-specific structure.

## Discussion

Using repeated cross-sectional CSLC data, we found that the proportion of adults reporting current diabetes care use rose from 1986 to 2022. Because this outcome reflects current care use rather than all diabetes, the rise reflects changes in underlying disease burden, diagnosis, treatment uptake, and care seeking. Educational differences were evident, particularly among working-age adults, whereas occupational differences were modest and non-monotonic. To our knowledge, this is the first nationally representative assessment of long-term trends in self-reported diabetes care use in Japan. The sex inequalities widened significantly across 1986–2022, driven largely by higher rates among men aged 50 years and older. Lower educational level, non-employment, advancing age, men, comorbid hypertension, depression, and smoking were key associated factors.

Our outcome captures only individuals currently under medical care for diabetes, excluding both diagnosed-but-untreated and undiagnosed cases. The 2024 NHNS reports that 26.9% of diagnosed men and 39.5% of diagnosed women were not in current treatment,^10^ and global estimates suggest undiagnosed cases account for a further 40–50% of the true burden.^19^ As underdiagnosis and undertreatment are likely greater in socioeconomically disadvantaged groups and younger adults,^20^ our estimates, if anything, understate the magnitude of the socioeconomic inequalities in the underlying disease burden.

A Japanese validation study comparing self-report against fasting glucose, HbA1c, and oral glucose tolerance testing reported sensitivity of 70.4%, with most missed cases having normal fasting glucose but abnormal HbA1c or postload glucose^21^; this subgroup is likely under-represented in our estimates. Nonetheless, self-reported care use is the only measure of long-term diabetes care use trends available from nationally representative Japanese data spanning 1986–2022, and its consistent measurement across 13 waves is a strength for trend analysis.

The prevalence of diabetes (HbA1c ≥6.5% or on treatment) increases steeply with age in Japan: the 2024 NHNS reports it rising from 1.6% in men aged 20–29 years to 26.0% in men aged 70 years and older, and from 1.3% to 16.5% in women over the same range.^10^ Similar age gradients are reported in South Korea and China, where about 30% of adults aged 65 years and older have diabetes versus single-digit prevalence in young adults.^22 23^ These patterns mirror our current findings on the diabetes care use presented in this study.

Diabetes prevalence and use of care strongly depend on SES. In high-income societies, lower SES is generally associated with a higher prevalence.^24^ We report similar findings in this study: adults with low and middle educational levels were 1.22 and 1.17 times as likely to be in diabetes care as those with high education. A recent large Japanese cohort study found that women in lower household income had 26% higher odds of diabetes compared with those in the highest income group after adjusting for age, family history, body mass index (BMI), and lifestyle factors.^25^ The inverse gradient was clearest among working-age adults and older women but less consistent among older men (65–94 years), where the middle-education group had the highest care use. This may reflect differential survival, cohort-specific educational composition, or shifting care patterns at older ages; we caution against attributing it to any single mechanism.

The SES-diabetes gradient is well documented in high-income countries. In the USA, adults living in poverty are about twice as likely to be diagnosed with diabetes as those with high incomes.^26^ A UK cohort found that lower lifelong SES significantly increased diabetes incidence in older adults,^27^ and a meta-analysis confirmed the SES gradient across countries.^24^ Underlying mechanisms are multifactorial: lower SES is associated with poorer diet quality, higher stress, and limited access to preventive care. Similar SES-related inequalities have also been observed in other East Asian countries. In China, urban residents had higher diabetes prevalence than rural residents (12.7% vs 11.1% in 2018), partly attributable to urban lifestyle risk factors.^23^ Wealthier regions in China had a greater diabetes burden as diets westernized, but over time, lower income segments also experienced higher rates as healthy food became less affordable and physical labor decreased.^23^ A South Korean analysis reported diabetes prevalence of 12.5% among adults 19 years and older in 2022, with higher rates among those with lower educational levels.^28^ Across East Asia, diabetes appears increasingly concentrated among lower SES groups.

Our analysis revealed one occupational finding that requires careful interpretation. While educational level showed a clear and consistent inverse gradient, the pattern for occupational class was more complex: lower non-manual workers had a lower prevalence (PR=0.92, 95% CI 0.86 to 0.98), while manual and farm workers showed no significant difference from upper non-manual workers (reference group). One possible explanation, which we cannot formally test with the present cross-sectional data, is the ‘healthy worker effect’,^29^ whereby individuals currently employed are selected for better health than the general population, which would compress differences in diabetes care use between occupational categories. The corresponding finding that the ‘non-employee’ group had a higher prevalence (PR=1.10, 95% CI 1.01 to 1.21) is broadly consistent with this possibility, since non-employees in this category likely include individuals who have left the workforce because of chronic illness. However, our cross-sectional design cannot disentangle selection into employment from other mechanisms, and alternative explanations cannot be ruled out. The divergence between educational and occupational findings suggests that education may serve as a more informative descriptive marker of socioeconomic inequality in this population than occupational class alone.

Our multivariable analysis identified several factors of self-reported diabetes care use. The strongest of these was comorbid hypertension (PR=1.79), consistent with the well-established clustering of cardiometabolic conditions: hypertension and type 2 diabetes share upstream risk factors (obesity, insulin resistance, physical inactivity) and are also clinically comanaged in Japanese primary care, meaning that individuals engaged with the care system for one condition are more likely to be diagnosed and treated for the other.^30 31^ Depression (PR=1.40) was also associated with higher prevalence, in line with bidirectional evidence that depression increases the risk of type 2 diabetes (through cortisol dysregulation, reduced adherence to healthy behaviors, and medication side effects), while a diabetes diagnosis itself elevates depression risk.^32^ Current smoking showed a modest positive association (PR=1.07), consistent with the established causal effect of smoking on type 2 diabetes incidence, although the effect size in our data is attenuated, likely because current smoking captures a contemporary behavior, whereas diabetes risk accumulates over decades of exposure and people who previously smoked (grouped with people who do not smoke in our analysis) retain elevated risk.

Two findings require careful interpretation because they run opposite to the expected direction of a causal effect. Alcohol consumption was associated with lower prevalence of diabetes care use (PR=0.75), a pattern inconsistent with experimental and prospective evidence that heavy alcohol intake increases type 2 diabetes risk. We attribute this inverse association primarily to reverse causality: clinical management of diabetes in Japan routinely includes counseling on alcohol reduction, so individuals currently receiving diabetes care are more likely to have been advised to reduce or cease drinking.^33^ Selection effects may also contribute, as individuals with severe complications or advanced age are less likely to be people who drink. Similarly, participation in annual health screening was associated with lower prevalence (PR=0.89). We interpret this as reverse causality, where individuals already under regular medical care for diabetes are less likely to separately attend a health screening program, since their diabetes monitoring substitutes for the general population screening pathway. Both findings illustrate a general caution in cross-sectional analyses of care-seeking populations, whereby the adjusted associations with behavioral and preventive factors can reflect consequences of the diagnosis rather than its causes.

A primary strength of this study is the use of the CSLC, a nationally representative survey using two-stage stratified random sampling based on the population census, with detailed educational and occupational data. The primary multivariable model used the most recent (2022) wave; however, key associations were stable across 2010–2022 in a sensitivity analysis (online supplemental eTable 2).

This rich socioeconomic detail information allowed for a more robust assessment of the relationship between these indicators and diabetes care use. However, several limitations apply. First, the outcome is self-reported current diabetes care use rather than epidemiological prevalence: undiagnosed and diagnosed-but-untreated cases are not captured, which is particularly relevant for interpreting socioeconomic inequalities since disadvantaged groups may be less likely to be diagnosed or remain engaged in care. The exclusion of undiagnosed and diagnosed-but-untreated cases has implications for the interpretation of the socioeconomic inequalities reported here. The 2024 NHNS reports that a substantial fraction of diagnosed adults are not currently in treatment, particularly among younger adults aged 30–49 years. If these patterns hold in our population, then the socioeconomic gradients we observe are likely conservative estimates of the underlying gradients in disease burden, rather than overestimates. The direction of this potential bias therefore reinforces rather than undermines our principal finding. Second, as noted in the Methods section, the CSLC does not distinguish type 1 from type 2 diabetes; however, Japanese national data indicate that type 1 diabetes accounts for approximately 6% of adults with treated diabetes^13^ and has an annual incidence of approximately 4.4 per 100 000 person-years.^14^ We therefore interpret findings in light of type 2 diabetes epidemiology. Third, the multivariable analysis was cross-sectional and restricted to 2022, so associations are adjusted cross-sectionally, not causally. Fourth, educational level was available only from 2010, limiting assessment over the full study period. Fifth, respondents with missing or non-classifiable occupations were handled differently in descriptive versus regression analyses, which may affect comparability. Sixth, BMI, diet, physical activity, income, family history, and region/urbanicity were unavailable, so residual confounding is likely. Finally, the CSLC lacks laboratory measures (fasting glucose, HbA1c), so the estimates are not directly comparable with biomarker-based prevalence.

## Conclusions

The proportion of adults reporting current diabetes care use increased from 1986 to 2022 in Japan. In the most recent survey wave, lower educational level was associated with higher current diabetes care use, particularly among working-age adults, while the educational gradient was less consistent among older men. These findings help inform continued surveillance of socioeconomic differences in diabetes diagnosis and care in Japan.

## Supplementary material

10.1136/bmjdrc-2026-006042 online supplemental file 1

## Data Availability

Data may be obtained from a third party and are not publicly available.
